# Mechanisms underpinning the permanent muscle damage induced by snake venom metalloprotease

**DOI:** 10.1371/journal.pntd.0007041

**Published:** 2019-01-29

**Authors:** Harry F. Williams, Ben A. Mellows, Robert Mitchell, Peggy Sfyri, Harry J. Layfield, Maryam Salamah, Rajendran Vaiyapuri, Henry Collins-Hooper, Andrew B. Bicknell, Antonios Matsakas, Ketan Patel, Sakthivel Vaiyapuri

**Affiliations:** 1 School of Pharmacy, University of Reading, Reading, United Kingdom; 2 School of Biological Sciences, University of Reading, Reading, United Kingdom; 3 Molecular Physiology Laboratory, Centre for Atherothrombotic and Metabolic Disease, Hull York Medical School, Hull, United Kingdom; 4 School of Pharmacy, University of Reading Malaysia, Johor, Malaysia; Instituto Butantan, BRAZIL

## Abstract

Snakebite is a major neglected tropical health issue that affects over 5 million people worldwide resulting in around 1.8 million envenomations and 100,000 deaths each year. Snakebite envenomation also causes innumerable morbidities, specifically loss of limbs as a result of excessive tissue/muscle damage. Snake venom metalloproteases (SVMPs) are a predominant component of viper venoms, and are involved in the degradation of basement membrane proteins (particularly collagen) surrounding the tissues around the bite site. Although their collagenolytic properties have been established, the molecular mechanisms through which SVMPs induce permanent muscle damage are poorly understood. Here, we demonstrate the purification and characterisation of an SVMP from a viper (*Crotalus atrox*) venom. Mass spectrometry analysis confirmed that this protein is most likely to be a group III metalloprotease (showing high similarity to VAP2A) and has been referred to as CAMP (*Crotalus atrox* metalloprotease). CAMP displays both collagenolytic and fibrinogenolytic activities and inhibits CRP-XL-induced platelet aggregation. To determine its effects on muscle damage, CAMP was administered into the tibialis anterior muscle of mice and its actions were compared with cardiotoxin I (a three-finger toxin) from an elapid snake (*Naja pallida*) venom. Extensive immunohistochemistry analyses revealed that CAMP significantly damages skeletal muscles by attacking the collagen scaffold and other important basement membrane proteins, and prevents their regeneration through disrupting the functions of satellite cells. In contrast, cardiotoxin I destroys skeletal muscle by damaging the plasma membrane, but does not impact regeneration due to its inability to affect the extracellular matrix. Overall, this study provides novel insights into the mechanisms through which SVMPs induce permanent muscle damage.

## Author summary

Snakebite is a major neglected tropical disease that affects thousands of people in the rural areas of developing countries. As well as the deaths, snakebites result in a significant number of disabilities including permanent loss of limbs that alter the lifestyle of the victims. Snake venom is a mixture of different proteins with diverse functions; one of these major protein groups present in viper venoms are metalloproteases that primarily induce muscle damage. The mechanisms behind the development of snakebite (metalloprotease)-induced permanent muscle damage are poorly studied. Here, we have purified a metalloprotease (CAMP) from the venom of the Western diamondback rattlesnake, and characterised its function in mice. To determine the actions of CAMP in the development of permanent muscle damage, it was injected into the muscle of mice in a parallel comparison with cardiotoxin I (from the venom of the Red spitting cobra). The effects of these proteins on muscle regeneration were analysed at 5 and 10 days after injection. The results demonstrate that through a combination of effects on the structural scaffolds surrounding the tissues, blood vessels and regeneration, CAMP significantly affects the muscles, thereby leading to permanent muscle damage.

## Introduction

Snakebite envenomation is a recently reinstated neglected tropical disease [1] that causes around 100,000 deaths annually [2, 3] and innumerable permanent disabilities predominantly on the rural population living in the lower income regions of the world [4–6]. The significant rate of mortality and morbidity occurs due to the difficulties associated with the treatment of snakebites [7], which vary depending on the species [8], geographical location [9], age of the offending snake [10, 11], the quantity of venom injected, correct diagnosis and mode of treatment [12]. Snake venoms contain proteins and small peptides with diverse functional effects [12]. Medically important snakes are generally found in two main families; Elapidae, a family with venoms mainly composed of smaller, neurotoxic proteins such as phospholipase A2 (PLA2) and three finger toxins, and Viperidae, a family with generally larger proteins such as serine and metalloproteases that primarily affect the cardiovascular and musculoskeletal systems. Snake venom serine proteases (SVSPs) mainly cause systemic envenomation effects such as the alteration of blood pressure, activation or inhibition of coagulation factors and degradation of fibrinogen [13, 14]. However, snake venom metalloproteases (SVMPs) primarily induce local envenomation effects such as swelling, necrosis and extensive tissue/muscle damage as well as the activation of certain coagulation factors and degradation of fibrinogen. SVMP-induced muscle damage is often difficult to treat due to the delay in obtaining appropriate medical treatment and poor outcome of anti-snake venom (ASV) treatment in the local tissues [15, 16]. Hence, extensive tissue damage is frequently treated by fasciotomy, a surgical procedure to remove the damaged tissues, cleaning the affected areas followed by skin graft or amputation of affected limbs or fingers when fasciotomy fails to suffice [7]. This results in permanent disabilities for victims, and significantly affects their socio-economic status following snakebites. For example, long term (persisting for over 13 years) musculoskeletal disabilities were found in over 3% of snakebite victims in a rural population of Sri Lanka and of these over 15% had to undergo amputations [17].

Skeletal muscle is composed of myofibres surrounded by the collagen-rich basement membrane. This tissue is imbued with a resident stem cell population called satellite cells (SCs), located under that basement membrane (BM), which are able to regenerate a functional tissue even after extensive damage [18]. The BM plays a key role in muscle repair by orientating the regenerating myofibres, a process mediated by SCs and acting as a scaffold for fibres to grow parallel to the existing fibres [19]. The majority of the direct myotoxic effects of venoms are attributed to PLA2 [20]. They can induce either local or systemic effects depending on their specificity to muscle cells (systemic effects) or a broader range of cells (local effects) through hydrolysis of phospholipids in plasma membrane. Other myotoxic venom components include sodium channel-blocking myotoxins [21] and muscle fibre depolarising cardiotoxins [22]. SVMPs are enzymatic proteins that primarily attack the collagenous structures and various other important components of BM to induce muscle damage. It has recently been reported that SVMPs induce haemorrhage by cleaving components of the BM and extracellular matrix surrounding the smaller blood vessels [23] although as multi-domain proteins, they are capable of binding to and cleaving a range of different proteins [24, 25].

SVMPs are generally classified into four groups based on the additional domains present in their structure: PI/Group I—contains only a metalloprotease domain; PII/Group II—contains a metalloprotease and disintegrin domain, and in some cases the disintegrin domain has been reported to be processed and liberated as a free disintegrin; PIII/Group III—contains a metalloprotease, a disintegrin-like and cysteine-rich domains; PIV/Group IV—contains two lectin-like domains connected by disulphide bonds to the other domains that are found in PIII SVMPs [26]. Although disintegrin-like domains show high sequence identity to disintegrins, they lack the typical RGD motif found in the venom disintegrins, which inhibit platelet aggregation via selectively blocking integrins. Both disintegrin-like and cysteine-rich domains have been found to inhibit collagen-induced platelet aggregation and induce early events of acute inflammation [27]. Notably, disintegrin-like domains were reported to contain an ECD motif that interacts with integrins and block their functions [28]. The non-proteinase domains play key roles in determining the diverse pharmacological effects of PII, PIII and PIV classes of SVMPs including the activation of coagulation factor X [29] and prothrombin [30] amongst others. These domains have also been found to co-localise in muscles, facilitating the hydrolysis of collagen and other BM components by the metalloprotease domain and promoting its accumulation in the BM [24], exerting haemotoxic activities. Moreover, SVMPs are also known to cause ischaemia in the local tissues due to poor blood supply as a result of their haemotoxic effects [31], which may prevent phagocytic removal of necrotic debris and reduce the supply of oxygen and nutrients needed for regeneration [32]. Given the complexity of their actions, a better understanding of the molecular mechanisms through which SVMPs induce permanent muscle damage may pave the way to the development of improved therapeutic strategies for snakebites. In this study, we demonstrate novel insights into the mechanisms by which a PIII/group III metalloprotease isolated from the venom of a North American viper, the western diamondback rattlesnake, *Crotalus atrox* triggers permanent muscle damage. Our results establish that this SVMP induces muscle damage and also prevents muscle regeneration by acting on the BM, myofibres, blood supply and SCs.

## Materials and methods

### Materials

Lyophilised *C*. *atrox* venom was purchased from Sigma Aldrich (UK) and the purified Cardiotoxin 1 (CTX), a three-finger toxin from the venom of *Naja pallida* was obtained from Latoxan (France).

### Protein purification

*C*. *atrox* venom (10mg) was dissolved in 1mL of 20mM Tris.HCl buffer (pH 7.6) and centrifuged at 5000g for 5 minutes before applying to a pre-made 1mL HiTrap™ Q HP Sepharose anion exchange column. Protein elution was performed at a rate of 1mL/min using 1M NaCl/20mM Tris.HCl gradient (up to 60%) by an ÄKTA purifier system (GE Healthcare, UK) over 20 minutes. The collected fractions were analysed by SDS-PAGE using standard protocols as described previously [33] and fractions with the protein of interest were pooled. The pooled fractions were then concentrated using a Vivaspin centrifugal filter and applied to a gel filtration column (Superdex 75, 1.6cm x 70cm). Protein elution was performed at a rate of 1mL/min using 20mM Tris.HCl (pH 7.6). Following SDS-PAGE analysis, the fractions containing the protein of interest were pooled and concentrated before running through the same gel filtration column again for further purification. Finally, the fractions containing the pure protein were pooled, concentrated and stored at -80°C until further use. Protein estimation was performed using Coomassie plus protein assay reagent (ThermoFisher Scientific, UK) and bovine serum albumin as standards.

### Mass spectrometry

The purified protein was subjected to SDS-PAGE, and a gel section containing the pure protein was subjected to tryptic digestion and analysed by mass spectrometry at AltaBioscience (Birmingham, UK). The extracted protein (10μg) from the gel slice was added to 100mM ammonium bicarbonate (pH 8). This was then incubated with dithiothreitol (10mM) at 56°C for 30 minutes. After cooling to room temperature, the cysteine residues were alkylated using iodoacetamide (50mM). Trypsin gold (Promega, UK) was subsequently added and the samples were incubated overnight at 37°C. The digested peptides were concentrated and separated using an Ultimate 3000 HPLC series (Dionex, USA). Samples were then trapped on an Acclaim PepMap 100 C18 LC column, 5um, 100A 300um i.d. x 5mm (Dionex, USA), then further separated in Nano Series Standard Columns 75μm i.d. x 15 cm. This was packed with C18 PepMap100 (Dionex, USA) and a gradient from 3.2% - 44% (v/v) solvent B (0.1% formic acid in acetonitrile) over 30 minutes was used to separate the peptides. The digested peptides were eluted (300nL/min) using a triversa nanomate nanospray source (Advion Biosciences, USA) into a LTQ Orbitrap Elite Mass Spectrometer (ThermoFisher Scientific, Germany). The MS and MS/MS data were then searched against Uniprot using Sequest algorithm and the partial sequence was then compared to the other similar protein sequences available in the protein database.

### Fibrinogenolytic assay

Human plasma fibrinogen (100μg/mL) was incubated with different concentrations of the whole venom or the purified protein, and a small volume of digested samples were removed at 30, 60, 90 and 120 minutes and mixed with reducing sample treatment buffer [4% (w/v) SDS, 10% (v/v) β-mercaptoethanol, 20% (v/v) Glycerol and 50mM Tris.HCl, pH 6.8]. The samples were then analysed by 10% SDS-PAGE and stained with Coomassie brilliant blue to determine the fibrinogenolytic activity of venom and the purified protein.

### Enzymatic assays

The metalloprotease activity of both *C*. *atrox* whole venom and the purified protein was assessed using a fluorogenic substrate, DQ-gelatin (ThermoFisher Scientific, UK). Briefly, the whole venom or purified protein (10μg/mL) was mixed in phosphate buffered saline (PBS, pH 7.4) with DQ gelatin (10μg/mL). The reaction mix was incubated at 37°C and the level of fluorescence was measured at 60 minutes using an excitation wavelength of 485nm and emission wavelength of 520nm by spectrofluorimetry (FLUOstar OPTIMA, Germany).

Similarly, the serine protease activity was measured using a selective substrate, Nα-Benzoyl-L-Arginine-7-Amido-4-methylcoumarin hydrochloride (BAAMC) (Sigma Aldrich, UK). The whole venom or the purified protein (10μg/mL) was incubated with BAAMC (2μM) at 37°C and the level of fluorescence was measured at an excitation wavelength of 380nm and emission wavelength of 440nm by spectrofluorimetry.

### Ethical statement

The University of Reading Research Ethics Committee has approved the procedures for blood collection from healthy human volunteers and the consent forms used to obtain written consent. Experiments with mice were performed in line with the principles and guidelines of the British Home Office and the Animals (Scientific Procedures) Act 1986 (PPL70/7516). All the procedures were approved by the University Research Ethics Committee (License number: UREC 17/17).

### Platelet aggregation

Human blood was obtained from healthy volunteers in vacutainers with 3.2% (w/v) sodium citrate as an anti-coagulant and the platelet-rich plasma (PRP) was prepared as described previously [34–36]. Platelet aggregation assays were performed by optical aggregometry using 0.5μg/mL cross-linked collagen related peptide (CRP-XL) as an agonist in the presence and absence of different concentrations of the purified protein.

### Administration of CTX or the purified protein in mice

The C57BL/6 mice (8 weeks old) were obtained from Envigo, UK. Mice were anaesthetised with 3.5% (v/v) isofluorane in oxygen before maintaining at 2% for the procedure. They were then injected intramuscularly with 30μL of either PBS (undamaged control), 50μM CTX, and 8 or 16 μM of the purified protein into their right tibialis anterior muscle. Mice were then allowed to recuperate for either 5 or 10 days before sacrificing by carbon dioxide asphyxiation and cervical dislocation.

### Dissection of tibialis anterior (TA) and extensor digitorum longus (EDL)

The TA muscles from mice were dissected, weighed and frozen on liquid nitrogen cooled iso-pentane prior to storage at -80°C. The EDL muscle was dissected from the undamaged contralateral hind limb of experimental mice and immediately placed in a 2mg/mL collagenase solution (Sigma Aldrich, UK) and incubated at 37°C with 5% CO_2_ for 2 hours to isolate the single fibres as previously described [37].

### Proliferation and migration of satellite cells

To determine the proliferation of SCs and myogenic differentiation, isolated single fibres were cultured for up to 48 hours at 37°C with 5% CO_2_ in single fibre culture medium (SFCM—DMEM, 10% (v/v) horse serum, 1% (v/v) penicillin-streptomycin and 0.5% (v/v) chick embryo extract) supplemented with either 0, 0.3, 1 or 3 μM of the purified protein prior to fixation in 2% (w/v) paraformaldehyde in PBS and maintained in PBS prior to immunocytochemistry.

The migration of muscle fibre SCs was analysed as described previously [37]. Briefly, the isolated single fibres were cultured for 24 hours in SFCM before transferring to SFCM containing 0, 0.3, 1 or 3μM of the purified protein and monitoring by a phase contrast microscope at 37°C with 5% CO_2_ using a 10X objective. A time-lapse video was captured at a rate of 1 frame every 15 minutes for a 24-hour period and analysed to determine the rate of migration.

### Immunohistochemistry

The collected TA muscles were mounted in Tissue-TEK^®^ OCT compound in an orientation allowing the transverse sections of 13μm thickness to be obtained using a cryo microtome. The tissue sections were incubated in permeabilisation buffer [20mM HEPES, 3mM MgCl_2_, 50mM NaCl, 0.05% (w/v) sodium azide, 300mM sucrose and 0.5% (v/v) Triton X-100] for 15 minutes at room temperature. To remove the excess permeabilisation buffer, 3 x 5 minute washes were performed using PBS before the application of wash buffer [PBS with 5% fetal bovine serum (v/v), 0.05% (v/v) Triton X-100] for 30 minutes at room temperature.

Primary antibodies were pre-blocked in wash buffer for 30 minutes prior to application onto muscle sections overnight at 4°C. In order to remove the primary antibodies, muscle sections were washed three times (5 minutes each) in wash buffer. The sections were then incubated with species-specific secondary antibodies that were conjugated with Alexa Fluor 488 or 594. The secondary antibodies were pre-blocked in wash buffer for minimum of 30 minutes before their application onto the slides and incubated for 1 hour in the dark at room temperature. Thereafter, the muscle sections were washed 3 x 5 minutes in PBS to remove the unbound secondary antibodies. Finally, the slides were mounted in fluorescent mounting medium, and the myonuclei were visualised using 4, 6-diamidino-2-phenylindole (DAPI) (2.5μg/mL). The images of sections were obtained using a fluorescence microscope (Zeiss AxioImager) and analysed using ImageJ. Macrophages were detected by F4.80 staining using the Vector Laboratories ImmPRESS Excel Staining Kit. A list of antibodies used in this study is provided in S1 Table.

### Statistical analysis

All the statistical analyses were performed using GraphPad Prism 7 and the P-values were calculated using one-way ANOVA followed by Dunnett’s post hoc multiple comparisons test.

## Results

### Protein purification and identification

**Fig 1:**
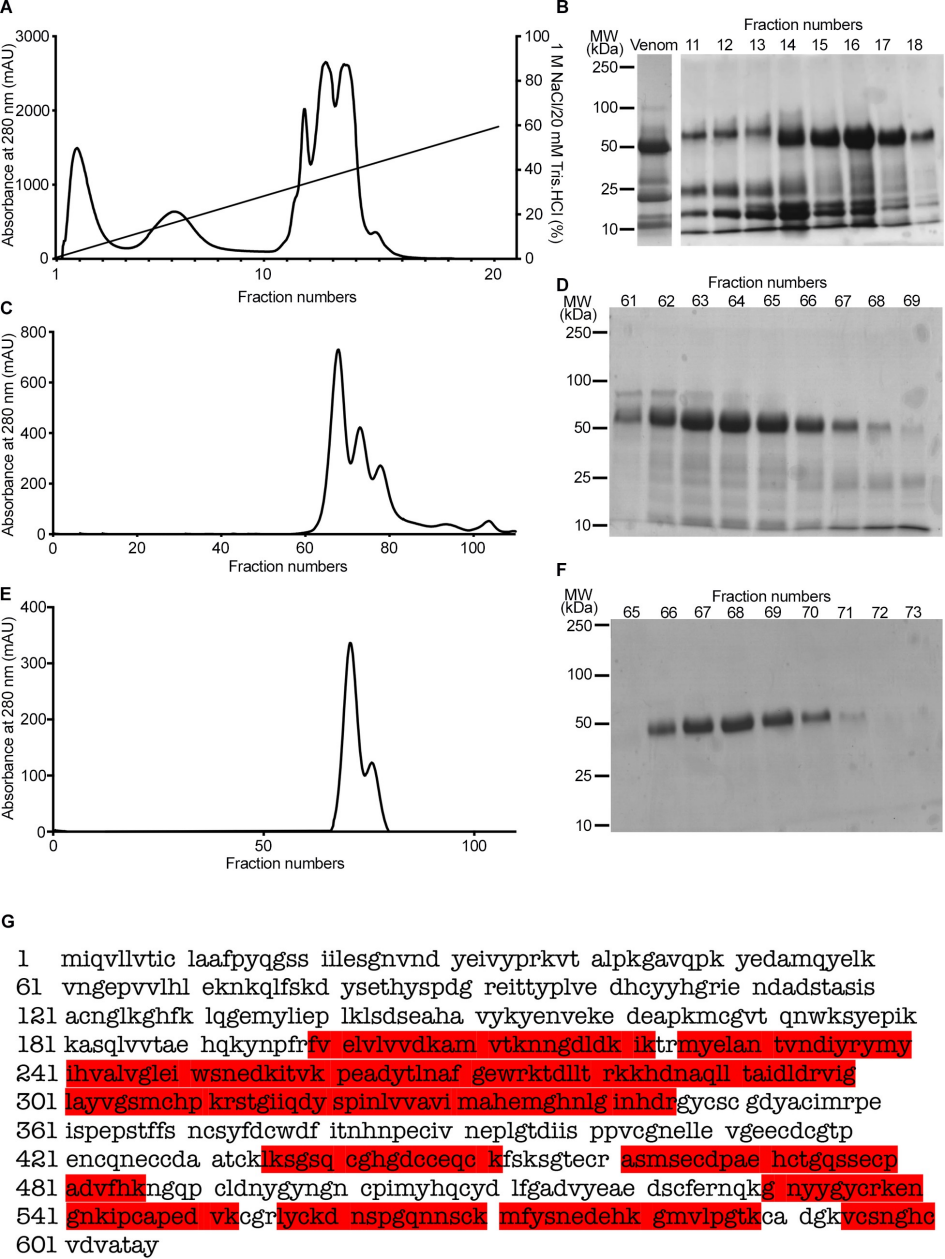
Purification of CAMP from the venom of C. atrox. **A**, A chromatogram demonstrates the purification profile of 10mg of whole *C*. *atrox* venom by anion exchange chromatography. **B,** a Coomassie stained gel displays the protein profile of whole *C*. *atrox* venom and fractions 11–18 of anion exchange chromatography. A chromatogram (**C**) and Coomassie stained gel (**D**) show the purification profile of fractions 14–18 of anion exchange chromatography by gel filtration. **E,** a chromatogram of the second step of gel filtration for fractions 62–67 from the previous step and (**F)** a Coomassie stained gel shows the purified protein at approximately 50kDa. **G,** the tryptic digested samples of the purified protein were analysed by mass spectrometry and the identified peptide sequences match (via Mascot search) with the known sequence of VAP2A (coverage 43%; the mass spectrometry-identified peptide sequences of the purified protein are shown in red) and confirms that the purified protein is most likely to be VAP, a group III metalloprotease. The purified protein was named as CAMP to represent *C*. *atrox* metalloprotease.

In order to purify a protein with a molecular weight of around 50kDa (as predicted for group III SVMPs) from the venom of *C*. *atrox*, a two-dimensional chromatography approach was employed. Following the initial fractionation of venom via anion exchange chromatography (Fig 1A and 1B), the selected fractions (14–18) with a highly abundant protein at approximately 50kDa were pooled and run through a gel filtration chromatography column (Fig 1C and 1D). The fractions (62–67) were pooled and run through the same gel filtration column again to refine the purification (Fig 1E and 1F). Finally, a pure protein with a molecular weight of around 50kDa was isolated. Mass spectrometry characterisation of the tryptic digested peptides of this protein and further Mascot analysis confirmed it to be a similar or identical protein to vascular apoptosis inducing proteins (VAP) such as VAP2, a protein with a molecular weight of 55kDa (an identical molecular weight to the purified protein) [38], which is a group III metalloprotease (Fig 1G). The identified peptide sequences of the purified protein covered around 43% of the sequence of VAP2A (highlighted in red in Fig 1G). The purified protein has been referred to as ‘CAMP’ to denote *C*. *atrox* metalloprotease throughout this article.

### Fibrinogenolytic and collagenolytic activities of CAMP

**Fig 2:**
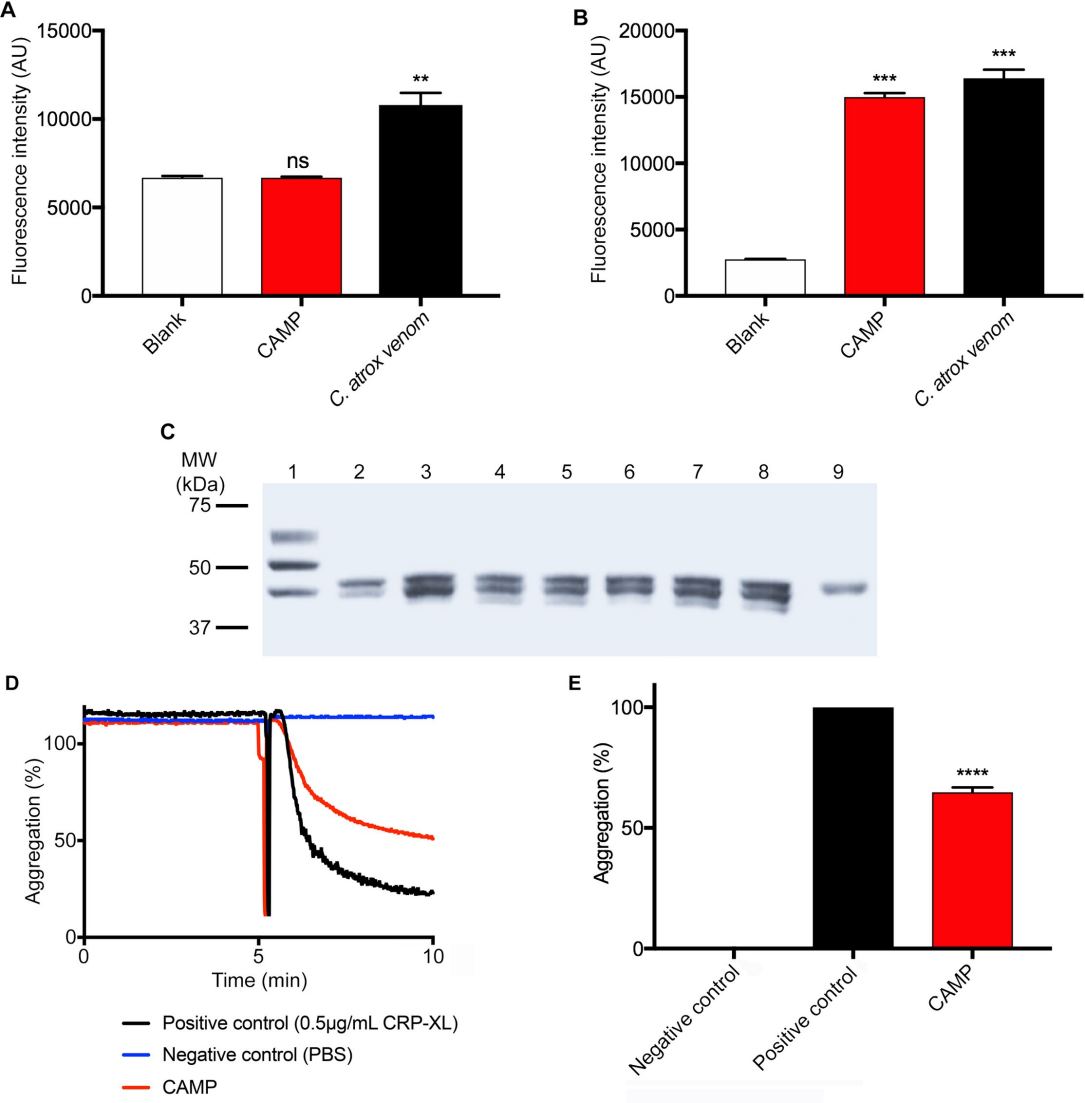
The functional characterisation of CAMP. **A**, the serine protease activity of 10μg/mL whole venom or CAMP was analysed using a fluorogenic substrate, Nα-Benzoyl-L-Arginine-7-Amido-4-methylcoumarin hydrochloride (BAAMC) by spectrofluorimetry. Similarly, (**B)** the metalloprotease activity of 10μg/mL whole venom or CAMP was analysed using DQ-gelatin, a specific fluorogenic substrate for collagenolytic enzymes and the level of fluorescence was measured by spectrofluorimetry. **C**, a Coomassie stained gel demonstrates the fibrinogenolytic activity of CAMP in comparison with whole *C*. *atrox* venom. Lanes, 1—undigested fibrinogen, 2—fibrinogen incubated with whole venom (100μg/mL), fibrinogen incubated with CAMP (100μg/mL) after 30 (3), 60 (4) and 90 (5) minutes, fibrinogen incubated with CAMP (50μg/mL) after 30 (6), 60 (7) and 90 (8) minutes, and CAMP alone (9). Representative aggregation traces (**D**) and data (**E**) demonstrate the impact of CAMP on cross-linked collagen related peptide (CRP-XL)-induced human platelet (PRP) aggregation. Data represent mean ± S.D. (n = 3). The *p* values shown are as calculated by One-way ANOVA followed by post hoc Tukey's test using GraphPad Prism (**p<0.01, ***p<0.001 and ****p<0.0001).

By using fluorogenic substrates, the protease activity of CAMP was analysed in comparison to the whole venom. CAMP displayed no serine protease activity as it failed to cleave a serine protease selective fluorogenic substrate, BAAMC although the whole venom displayed significant serine protease activity (Fig 2A). However, it showed high levels (similar to the whole venom) of collagenolytic activity (Fig 2B). Furthermore, the ability of CAMP to digest fibrinogen was analysed by incubating it with human plasma fibrinogen. The SDS-PAGE analysis of samples that were taken at different points of incubation confirmed that CAMP is capable of cleaving Aα and Bβ chains of fibrinogen although it was unable to cleave the γ chain (Fig 2C). The digestion of fibrinogen with CAMP appears to be rapid as the levels of Aα and Bβ chains of fibrinogen were reduced significantly as early as 30 minutes of incubation. These results corroborate CAMP as an SVMP with collagenolytic and fibrinogenolytic activities, which may affect the collagen in the BM around the local tissues at the bite site and fibrinogen in the blood.

### CAMP inhibits human platelet aggregation

The ability of CAMP to inhibit agonist-induced platelet activation was analysed using human platelet-rich plasma (PRP) by optical aggregometry. The pre-treatment of human platelets (PRP) with CAMP (50μg/mL) has significantly inhibited 0.5μg/mL CRP-XL-induced platelet aggregation (Fig 2D and 2E). This data confirms the ability of CAMP to affect human platelet activation.

### CAMP induces haemorrhage and fluctuations in muscle size in mice

**Fig 3:**
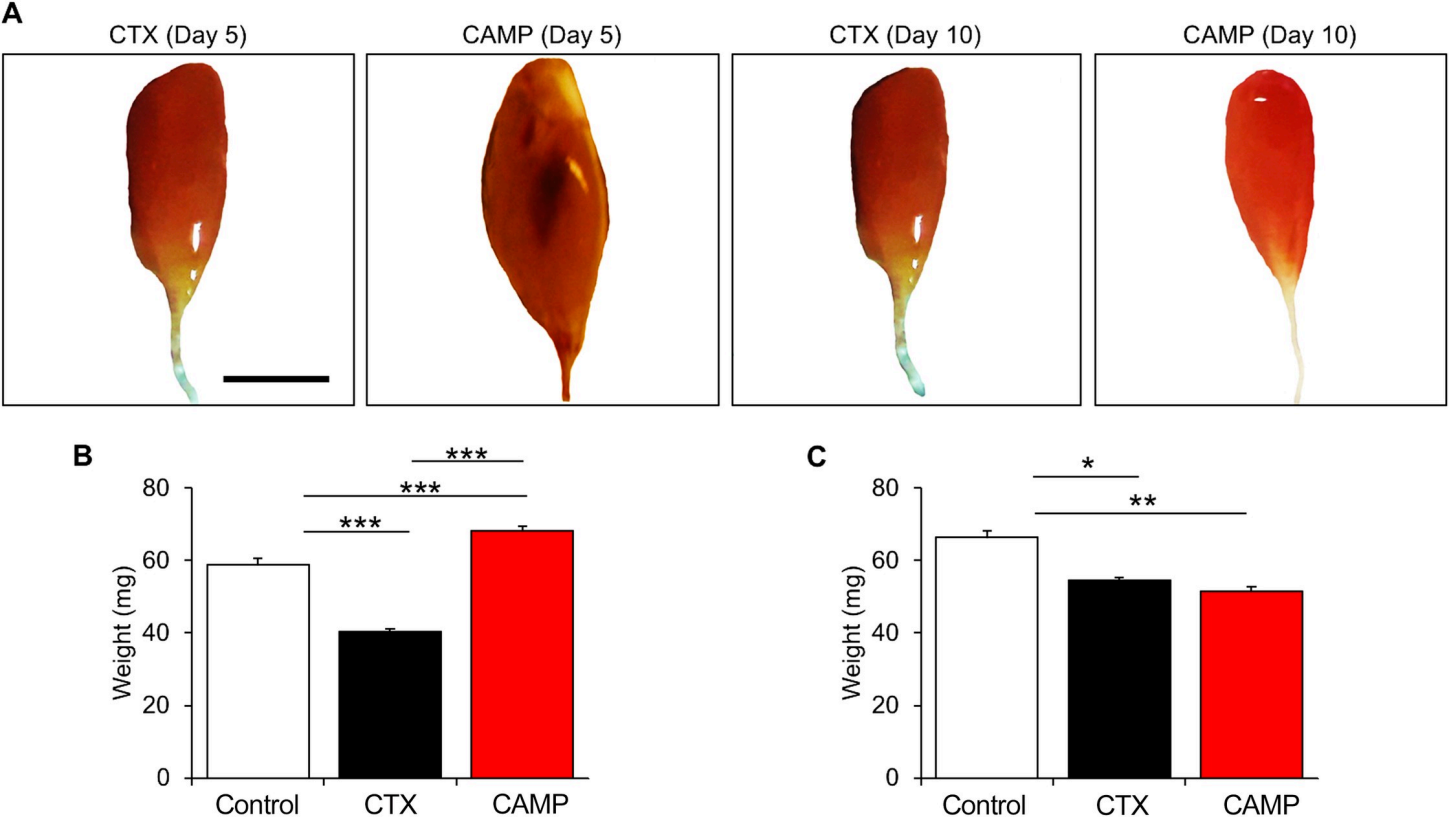
Macrostructure of tibialis anterior muscle after CAMP treatment. **A**, representative images of muscles treated with either CTX or CAMP for 5 or 10 days. Quantification of TA weight at 5 (**B**) and 10 (**C**) days post administration. Scale bar represents 5 mm. Data represent mean ± S.D. (n = 5 for each cohort). The *p* values shown are as calculated by One-way ANOVA followed by post hoc Tukey's test using GraphPad Prism (*p<0.05, **p<0.01 and ***p<0.001).

In order to determine the mechanisms through which SVMPs induce permanent muscle damage, CAMP was used as a tool to determine its pathological effects in TA muscle of mice in comparison with CTX. The intramuscular injection of CAMP induced haemorrhage in the damaged muscles and thereby, caused swelling and increase in muscle weight after five days of administration (Fig 3A and 3B). However, CTX did not induce haemorrhage or swelling although the muscle weight was reduced compared to the controls at the same time point. In contrast, after ten days of administration, muscle weight in CAMP-treated mice was decreased similar to CTX-treated muscle (Fig 3A and 3C). These data demonstrate that CAMP is capable of inducing haemorrhage and swelling and thereby, increases in muscle weight initially although it decreases at a later time point.

### Attenuated regeneration in CAMP-damaged muscle

**Fig 4:**
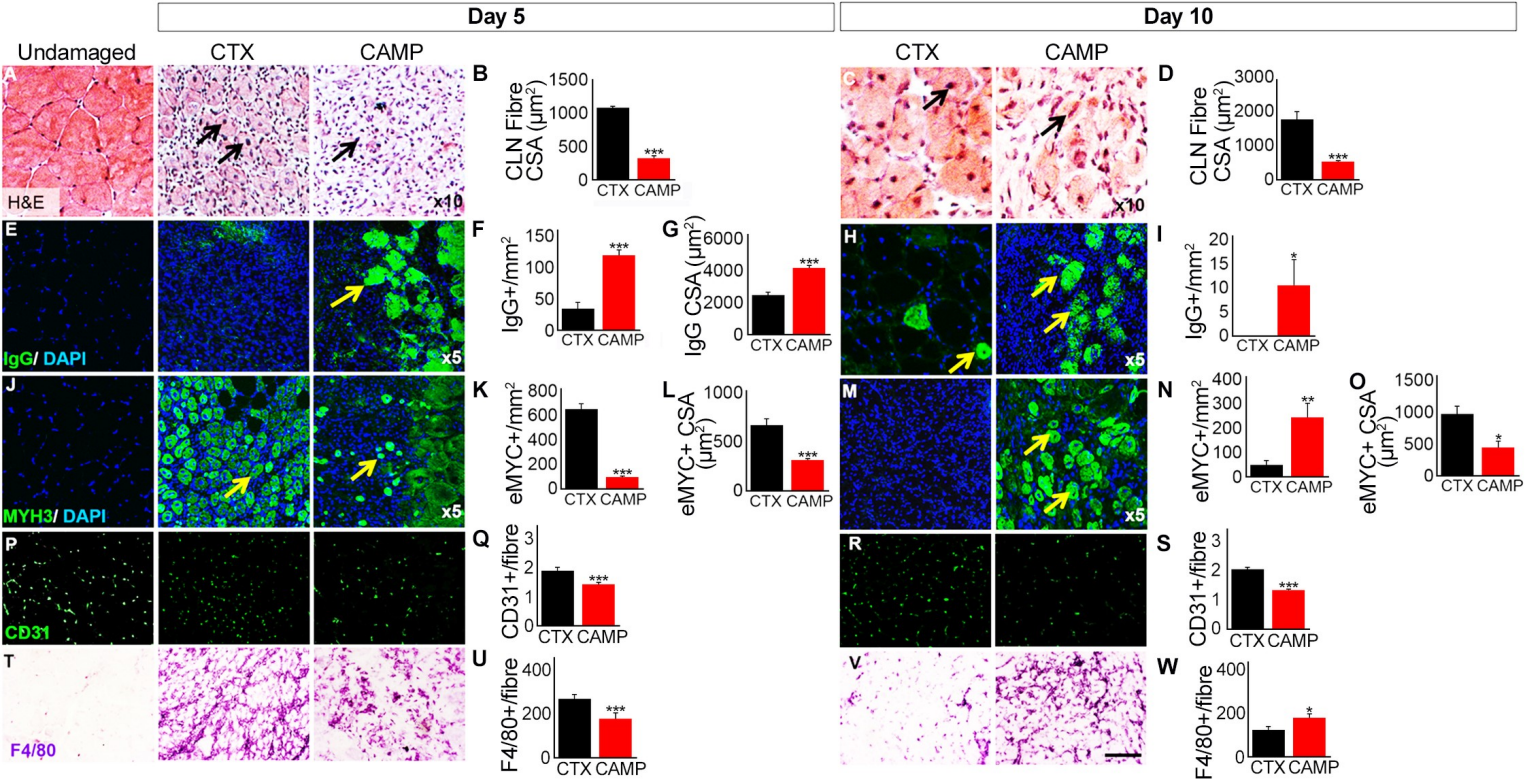
Analysis of tibialis anterior muscle regeneration after administration of CAMP or CTX. **A,** H and E staining of muscle identifying centrally located fibre nuclei (CLN) (arrows) and (**B**) quantification of centrally located muscle fibre size 5 days post administration. **C,** H and E staining of muscle (arrows) and (**D**) quantification of centrally located muscle fibre size 10 days post administration. **E**, intra-fibre IgG localisation for necrotic muscle fibres (arrows) and quantification of necrotic fibre density **(F)** and size (**G**) 5 days post administration. **H**, intra-fibre IgG localisation for necrotic fibres (arrows) and (**I**) quantification of necrotic fibre density 10 days post administration. **J,** identification of regenerating muscle fibres through the expression of MYH3 (arrows) and quantification of regenerating muscle fibre density (**K**) and size (**L**) 5 days post administration. **M,** identification of regenerating muscle fibres through the expression of MYH3 (arrows) and quantification of regenerating muscle fibre density (**N**) and size (**O**) 10 days post administration. **P,** Localisation of endothelial marker CD31 and **(Q)** quantification of capillaries per regenerating muscle fibre 5 days post administration. **R,** localisation of endothelial marker CD31 and **(S)** quantification of capillaries per regenerating muscle fibre 10 days post administration. **T,** immunostaining with antibody F4/80 and **(U)** its density quantification in damaged region 5 days post administration. **V,** immunostaining with antibody F4/80 and **(W)** its density quantification in damaged region 10 days post administration. Data represent mean ± S.D. (n = 5 for each cohort). The *p* values shown are as calculated by two-tailed Student’s T test for independent variables using GraphPad Prism (*p<0.05, **p<0.01 and ***p<0.001).

We examined the cellular processes underpinning the morphology of skeletal muscle and assessed muscle regeneration after damage induced by CAMP. Haematoxylin (H) and eosin (E) staining facilitates the identification of cellular organisation within a tissue and also the presence of fibres containing centrally located nuclei (CLN), which is an indicator of muscle regeneration. Five days after tissue damage, muscles treated with CTX contained many large fibres with CLN (Fig 4A). Furthermore, there were regions of high cell density between fibres displaying CLN. In contrast, 5 days after CAMP damage the number of fibres with CLN was less abundant and smaller than in CTX damaged muscle (Fig 4A and 4B). Additionally, there were areas of sparsely populated regions between fibres. Ten days after CTX damage, large fibres with CLN were evident with very little space between muscle fibres (Fig 4C and 4D). The fibres appeared to be regular in terms of shape and size, evidencing robust muscle regeneration. Whereas, at the same time point, muscle damaged with CAMP displayed smaller fibres with CLN and inter-fibre regions populated with cells were prominent (Fig 4C and 4D). Next, we documented the profile of dying muscle fibres, facilitating the infiltration of circulating immunoglobulins (Ig) into the damaged fibres. Five days after CTX injection, low density of small calibre fibres displayed the infiltration by IgG (Fig 4E–4G). In contrast, at the same time point, CAMP treatment resulted in not only a higher density of fibres with infiltrated IgG, but they were also of larger size (Fig 4E–4G). By day 10, very few dying fibres were present in CTX treated muscle, however dying fibres were prominent in CAMP treated muscles (Fig 4H and 4I). We then examined the presence of regenerating muscle fibres, facilitated through the expression of embryonic myosin heavy chain protein (MYH3). Muscle regeneration was clearly evident in muscles damaged by CTX at day 5 (Fig 4J and 4K). Large numbers of evenly sized fibres expressing MYH3 featured in CTX-damaged tissue (Fig 4J and 4L). In contrast to CTX treatment, the number of regenerating fibres in CAMP-treated muscle was lower and when present were of heterogeneous size (Fig 4J–4L). By Day 10, the expression of MYH3 has been cleared in CTX-damaged muscle and when present was in very large fibres (Fig 4M–4O). In contrast, MYH3 expression was clearly evident at day 10 in CAMP-damaged muscle but in smaller, non-uniform fibres (Fig 4M–4O). Next, we examined the impact of CAMP and CTX on blood vessels through immunostaining with the endothelial cell specific antibody, CD31. At both 5 and 10 days, the number of capillaries serving each regenerating fibre was greater in the CTX treated sample compared to CAMP (Fig 4P–4S). Importantly, the number of capillaries serving each regenerating fibre in the CTX treated sample was identical to the undamaged sample. Moreover, the degree of macrophage infiltration into the damaged area was analysed, as these cells are key to effective muscle regeneration. The density of macrophages in damaged muscle was greater in the CTX treated muscle compared to CAMP at day 5 (Fig 4T and 4U). However, by day 10, the situation was reversed; there was a greater density of macrophages in the CAMP treated samples compared to CTX (Fig 4V and 4W).

### CAMP extensively damages the extracellular matrix (ECM) surrounding the myofibres

**Fig 5:**
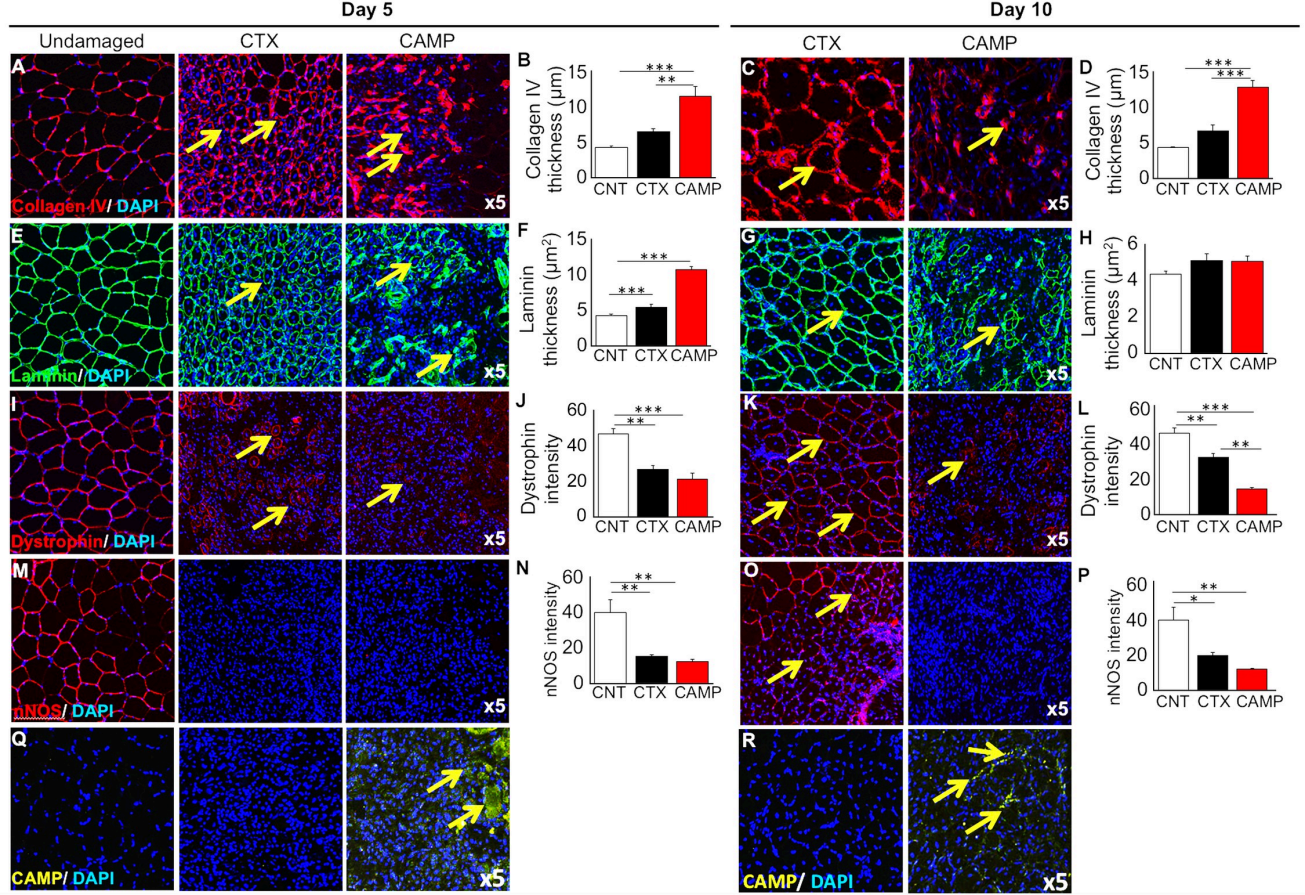
Immunohistochemical analysis of muscle extracellular matrix and associated proteins after tibialis anterior damage with CAMP or CTX. Localisation (arrows) (**A**) and thickness (**B**) of collagen IV 5 days post administration. Similarly, localisation (arrows) (**C**) and thickness (**D**) of collagen IV 10 days post injury. *Note*: CTX damaged tissues show circumferential collagen compared to foci in CAMP treatment. Localisation (arrows) (**E**) and thickness (**F**) of laminin 5 days post injury. Localisation (arrows) (**G**) and thickness (**H**) of laminin 10 days post injury. **I**, localisation of dystrophin 5 days post injury (arrows). *Note*: dystrophin around centrally located nuclei in CTX-treated muscle. In contrast, incomplete dystrophin domain around CAMP-damaged muscle. **J**, intensity of dystrophin 5 days post injury. Localisation (arrows) (**K**) and intensity (**L**) of dystrophin 10 days post injury. **M**, localisation of nNOS 5 days post injury (no circumferential nNOS was detectable at day 5) and (**N**) thickness of nNOS 5 days post injury. **O**, localisation of nNOS 10 days post administration (arrows). Note: circumferential nNOS was only detectable in CTX-treated muscle and (**P**) intensity of nNOS 10 days post injury. Localisation of CAMP in damaged region at day 5 (**Q**) and 10 (**R**) (arrows). Data represent mean ± S.D. (n = 5 for each cohort). The *p* values shown are as calculated by One-way ANOVA followed by post hoc Tukey's test using GraphPad Prism (*p<0.05, **p<0.01 and ***p<0.001). CNT represents control.

Efficient regeneration of skeletal muscle following acute damage is contingent on stem cells capable of replacing damaged tissue and their highly ordered formation into myotubes/fibres, a process orchestrated by the ECM. The organisation of collagen IV, a major BM component of muscle fibres was analysed as described previously [39]. A thin circle of collagen IV surrounding muscle fibre was evident 5 days after CTX treatment (Fig 5A). In contrast, at an identical time after CAMP treatment, muscle displayed large irregular, thick depositions of collagen IV (Fig 5A and 5B) but by day 10, the picture was even more polarised, as CTX-damaged muscle showed a relatively normal distribution of collagen IV (Fig 5C and 5D). In contrast, very few fibres from CAMP-treated muscle (at day 10) displayed a ring of collagen IV, and instead this protein was localised at thick foci (Fig 5C and 5D). A near-identical pattern was documented for the distribution of laminin, another major component of the muscle fibre ECM (Fig 5E–5H).

Furthermore, the impact of CTX and CAMP on molecules that are associated with linking the contractile apparatus to the ECM was investigated. Dystrophin is normally localised under the sarcolemma of mature muscle fibres. Its expression was evident around some of the larger regenerating muscle fibres 5 days after CTX damage (Fig 5I). Whereas, very few fibres expressing dystrophin were detected at a similar time point in CAMP-treated muscles (Fig 5I). However, when present, the thickness of the dystrophin expression domain was similarly reduced by the two treatments (Fig 5J). At day 10, most of the fibres from CTX-treated muscle displayed a continuum of dystrophin expression, although at a lower thickness compared to undamaged tissue (Fig 5K and 5L). However, very few fibres with a ring of dystrophin were present in CAMP-treated muscles at day 10 (Fig 5K). Furthermore, the domain, when present was thinner than both control as well as CTX treated muscles (Fig 5L). Then, the distribution of nNOS, a protein that localises to a sub-sacrolemmal position which is dependent on its binding to dystrophin was assessed. At 5 days after treatment, very little nNOS was present in either CTX or CAMP-damaged muscles (Fig 5M and 5N). By day 10, a thin band of nNOS was evident in CTX-treated muscle but not in the muscle damaged by CAMP (Fig 5O and 5P). Lastly, the muscles were analysed to determine the presence of remaining CAMP in damaged tissues. The immunohistological profiling showed that CAMP was clearly present at both 5 and 10 days after its administration (Fig 5Q and 5R). These results show that CAMP treatment damages not only the ECM of muscle fibres but also affects intracellular components that link it to the contractile machinery.

### CAMP affects the functions of satellite cells (SCs)

**Fig 6:**
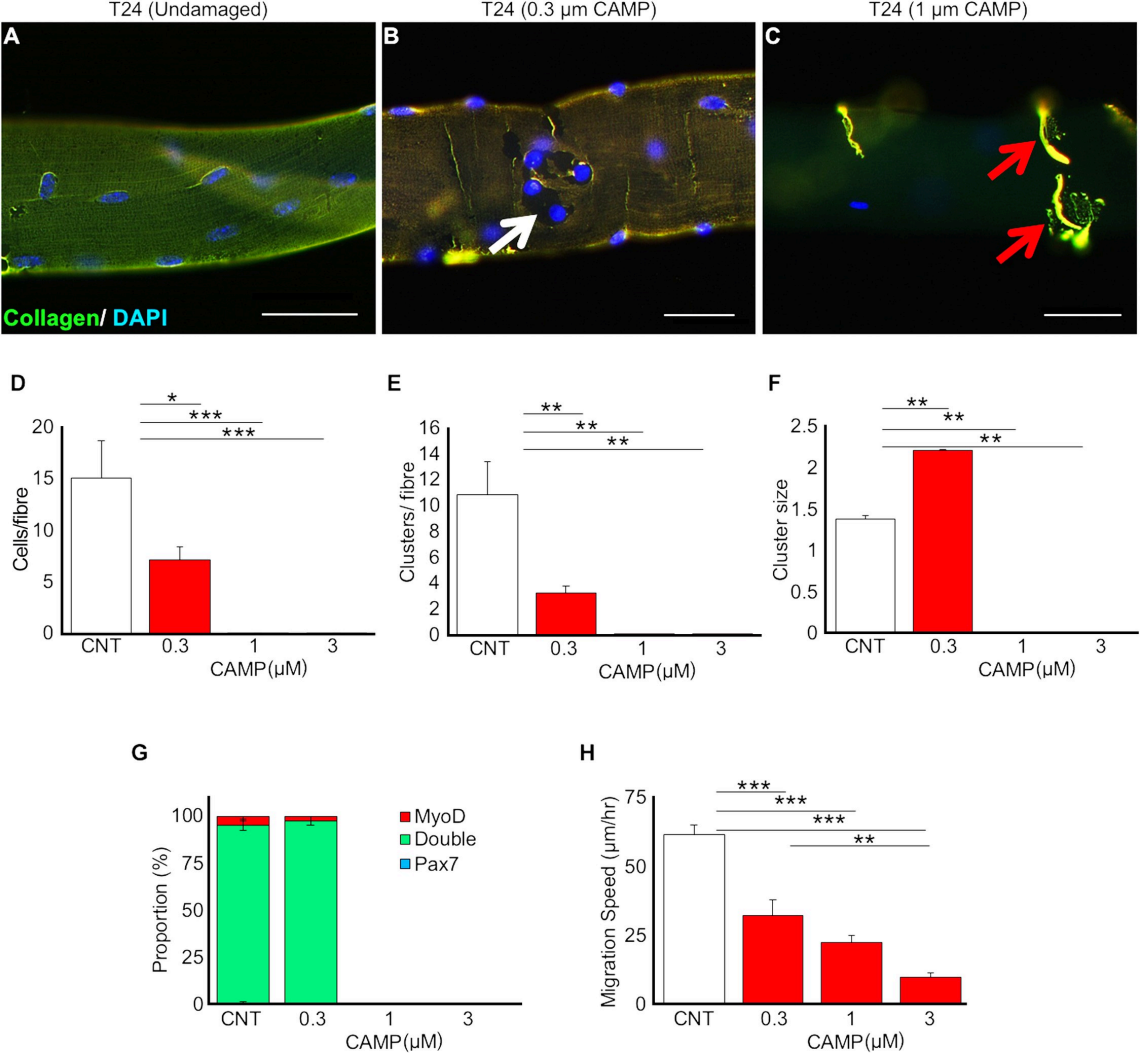
CAMP treatment affects proliferation and migration of satellite cells. (**A**-**C**) Increasing concentrations of CAMP change the distribution of collagen IV. Satellite cell proliferative characteristics following CAMP treatment were analysed. For example, satellite cell number per fibre (**D**), satellite cell clusters per fibre (**E**) and cluster size per fibre (**F**) were quantified. **G,** myogenic characteristics of satellite cells following CAMP treatment. **H**, migration rate of satellite cells. Scale bar represents 20μm. Data represent mean ± S.D. (n = 20 fibres for each cohort). The *p* values shown are as calculated by One-way ANOVA followed by post hoc Tukey's test using GraphPad Prism (*p<0.05, **p<0.01 and ***p<0.001). CNT represents control.

The role of SCs adjacent to the muscle fibres is critical for muscle regeneration. In order to determine the impact of CAMP on SCs, we have isolated myofibres from intact EDL muscles and exposed them to a range of concentrations of CAMP. As early as 24 hours after CAMP treatment, it was evident that there was a concentration dependent disturbance to the collagen component of the ECM around muscle fibres. A uniform layer of collagen expression was detected in untreated fibres (Fig 6A). At the lower concentration, CAMP caused a localised denuding of the myofibre (Fig 6B), whereas the higher concentration resulted in the absence of collagen from most parts of the fibres and caused it to concentrate in specific locations (Fig 6C). The cell growth, proliferation and migration were monitored on the isolated single muscle myofibres over a 48 hour time period. SCs were immunostained using the myogenic transcription factors, Pax7 (uncommitted cells) and MyoD (activated cells) in order to monitor the progression of cells through myogenesis. The concentrations of above 0.3μM of CAMP induced hypercontraction, which is indicative of extensive fibre damage. At 0.3μM, viable fibres were present, and revealed that CAMP significantly decreased the number of associated SCs (Fig 6D). Furthermore, the number of SC clusters was reduced per fibre (Fig 6E), although each cluster at the lower concentration had more cells than untreated fibres (Fig 6F). Analysis of differentiation was only possible at the lowest concentration of CAMP (Fig 6G) as at higher concentrations, hypercontraction prevented this analysis. The migration speed was calculated between 24 and 48 hours and was found to decrease significantly as the concentration of CAMP increased (Fig 6H). These data demonstrate that CAMP is able to affect both the proliferation and migration of SCs but not the differentiation.

## Discussion

The swelling and necrosis at the bite site as well as permanent muscle damage are common effects of snakebite envenomation (particularly viper bites). These effects frequently lead to amputation and therefore disable victims, which adds to their inability to earn money, and exacerbates the poverty that is already experienced by the vast majority of snakebite victims [4]. Here we have purified a metalloprotease from one of the most studied venomous snake species, *C*. *atrox*. Although deaths from this snake are now uncommon, disfigurement is still a prevalent side effect for survivors. SVMPs are a predominant component in viper venoms that are involved in inducing the local envenomation effects including muscle damage. The ability of SVMPs to degrade collagen has been established, but its impact on permanent muscle damage under *in vivo* settings has not been previously demonstrated in sufficient detail. Therefore, we deployed a metalloprotease from the venom of *C*. *atrox* and analysed its impact on skeletal muscle damage in comparison to a three-finger toxin, CTX from the venom of *Naja pallida*. Mass spectrometry analysis of the purified protein suggests it to be a group III metalloprotease, which possess a metalloprotease domain as well as a disintegrin-like and cysteine-rich domains [40]. Based on the peptide sequences identified by the mass spectrometry, the purified protein is likely to be VAP2 or one of its heterodimers; VAP2A or VAP2B, both of which are vascular apoptosis inducing proteins (38) that are known to be haemorrhagic [41] and in the case of VAP2B to inhibit collagen-induced platelet activation [42]. Due to the limited peptide sequences identified by mass spectrometry for the purified protein, we are unable to conclude whether the purified protein (CAMP) is identical to VAP2 or either of its heterodimers. CAMP was characterised to be a collagenolytic and fibrinogenolytic enzyme. It also inhibited CRP-XL-induced platelet aggregation; group III metalloproteases are known to interact with the integrin α_2_β_1_, binding to the α_2_ subunit and causing the shedding of β_1_ subunits [43]. However, VAP2B (a protein described from *C*. *atrox*) has been reported to inhibit collagen induced platelet aggregation by binding to collagen [44], although whether the SECD sequence found in disintegrin-like domains is able to bind CRP-XL in the same way, is unknown.

In order to determine the impact of SVMPs in stimulating permanent muscle damage, different concentrations of CAMP were administered in mice along with CTX and control groups and the effects were analysed at five and ten days after the administration. We suggest that this occurs at two levels; by breaking down the ECM which normally acts as a scaffold for the formation of new muscle fibres and around existing blood vessels and secondly attenuating properties of resident stem cells that are essential to effective tissue repair. SVMPs are known for their collagenolytic activities and for targeting various components of the BM in the vasculature and inducing haemorrhage [45]. In line with previous studies, here we demonstrate that CAMP induces haemorrhage and affects the architecture of collagen and laminin. The destruction of the collagen based ECM may be the key to long-term tissue destruction wrought by CAMP. The disintegrin-like and cysteine-rich domains have already been identified as essential to the haemorrhagic activity and ECM degradation attributed to the PIII metalloproteases [46]. This is in contrast to myotoxic PLA2 and three-finger toxins that are well documented in causing membrane permeabilisation and consequentially myonecrosis via the hydrolysis of membrane phospholipids or imbedding directly into the membrane respectively [47–49]. Our data emphasise that CAMP in comparison to CTX significantly hindered the regeneration of skeletal muscle fibres most probably by disturbing the organisation of the ECM. The elevated levels of necrosis seen five days after administration with CAMP improved after ten days, although it was still evident. However, it must be noted that at day 10, the CTX treated muscles had almost completely regenerated with healthy fibres. Moreover, very low levels of MYH3 were detected five days after CAMP treatment. In CTX-treated muscles, this marker of regenerating fibres was clearly evident and present at a high level. This indicates that the initiation of the regeneration process was attenuated by CAMP in comparison to CTX. Muscle regeneration is dependent on blood supply and clearance of damaged fibres. We show here that both these cellular compartments are affected in a detrimental manner by CAMP. We found that the number of capillaries serving each regenerating muscle fibre was smaller in CAMP treated muscle compared to CTX. Importantly the number of capillaries serving each fibre in damaged CTX muscle was the same as in undamaged regions. These results show that capillaries as well as muscle fibres are damaged by CAMP whereas it is only the latter in CTX treated tissue. Additionally, we show that there was a greater influx of macrophages into the CTX damaged muscle compared to regions affected by CAMP. Furthermore, the density of macrophages decreased in CTX treated muscle over time, attesting to regeneration. In contrast, the density of macrophages in CAMP treated muscle was lower at day 5 compared to CTX, possibly indicating an attenuated clearance process. Importantly the density of macrophages did not change in the CAMP treated muscle over 10 days suggesting on-going muscle damage. Although the abundance of MYH3 increased in CAMP-treated muscles by day 10, its expression in CTX-injured muscles was almost undetectable, signifying advanced regeneration. This was reflected in the appearance of dystrophin and nNOS at their normal sub-sarcolemmal position. In keeping with the notion that CAMP treatment not only affects the degree of regeneration but also its timing, we showed that very few fibres expressed dystrophin in its normal position and a significantly reduced expression of nNOS was observed even at day 10. Most importantly we show that CAMP is still present at the site of injury even 10 days after its administration and that it profoundly disorganises the ECM.

The single fibre experiments highlight another aspect to explain the attenuated muscle regeneration following CAMP-mediated muscle damage. We demonstrate that the proliferation and migration of SCs was significantly reduced by CAMP treatment. Both of these factors are key in promoting muscle regeneration. Therefore, CAMP may bring about permanent impairment of muscle organisation and function by firstly destroying muscle fibres, secondly breaking down the organisation of the ECM. This is required by the SCs in order to align and fuse in a coordinated manner and lastly by diminishing the ability for SCs to expand their numbers and migrate to the site of injury to enact efficient regeneration. It is clear that current ASV treatment is not effective at preventing muscle damage. Although translating the results of this study into therapeutics might be difficult, these will improve the understanding of SVMP-induced permanent muscle damage. The ability of group III metalloproteases to bind components of the BM and prolong muscle exposure to their myotoxic effects suggests a therapeutic agent that is capable of interacting with these enzymes and non-enzymatic domains and preventing the longevity of these proteins in the area surrounding fibres may be able to speed up the rate of regeneration considerably. Moreover, any drugs aimed at treating this aspect of snakebite envenomation may struggle to reach it intravenously, and therefore, they may have to be administered via multiple local injections considering the widespread damage to microvasculature [32] and consequential lack of blood supply to affected tissues.

ASV is the only effective treatment for systemic envenoming, however local venom pathology is largely unaffected by ASV when treatment is not immediately administered [50]. ASV is composed of large immunoglobulins that appear to struggle to reach the areas affected by SVMPs. The combination of small vessel destruction combined with BM cleavage results in a poor blood supply and therefore weak neutralisation by intravenously administered ASV. Local injections of ASV have also been found to be of no benefit to the snakebite victims [51]. However, there are a range of matrix metalloprotease inhibitors that have undergone testing for their specificity to SVMPs and some promising compounds have been identified [52]. The small molecule inhibitors aimed at the metalloprotease domain such as batimastat [53] have been tested extensively and they were found to abrogate the haemorrhagic effects of venom if administered immediately after envenoming. Given their haemorrhagic effects are largely dependent upon collagen degradation, it is reasonable to postulate that this prevention of haemorrhagic effects may also apply to muscle damage. Moreover, metal chelating agents such as EDTA have also been tested *in vivo* at non-toxic doses and found to prevent venom-induced lethality [54].

The need for immediate administration is of course unrealistic with conventional ASV but small stable inhibitors have the potential to be spread and made available to those in areas with a high density of snakebites. Multiple local injections do bring the potential for delivery directly to the bite site and administering to multiple sites may overcome the problematic spread of drug through a site of damaged muscles and vessels. Future experiments should aim to investigate the effect of these drugs on BM components, using both pre-incubation with drugs and post envenomation delivery models. Overall, the complete destruction or loss of a range of BM and dystrophin-glycoprotein complex components as well as the effect of this SVMP on muscle regeneration highlights the significant difficulties involved in treating the necrosis and muscle damage associated with snakebite envenomation. Hence, this study provides greater insights into the understanding of SVMP-induced permanent muscle damage and local snakebite envenomation effects.

## Supporting information

S1 Table The list of antibodies used in the immunohisto- and cytochemistry analyses. (DOCX)

## References

[1] WHO. Neglected tropical diseases 2017 [Available from: http://www.who.int/neglected_diseases/diseases/en/.

[2] Kasturiratne A, Wickremasinghe AR, de Silva N, Gunawardena NK, Pathmeswaran A, Premaratna R, et al The global burden of snakebite: a literature analysis and modelling based on regional estimates of envenoming and deaths. PLoS Med. 2008;5(11):e218 10.1371/journal.pmed.0050218 18986210 PMC2577696

[3] Chippaux JP. Snake-bites: appraisal of the global situation. Bulletin of the World Health organization. 1998;76(5):515 9868843 PMC2305789

[4] Harrison RA, Hargreaves A, Wagstaff SC, Faragher B, Lalloo DG. Snake envenoming: a disease of poverty. PLoS neglected tropical diseases. 2009;3(12):e569 10.1371/journal.pntd.0000569 20027216 PMC2791200

[5] Chippaux J-P. Estimate of the burden of snakebites in sub-Saharan Africa: a meta-analytic approach. Toxicon. 2011;57(4):586–99. 10.1016/j.toxicon.2010.12.022 21223975

[6] Vaiyapuri S, Vaiyapuri R, Ashokan R, Ramasamy K, Nattamaisundar K, Jeyaraj A, et al Snakebite and its socio-economic impact on the rural population of Tamil Nadu, India. PloS one. 2013;8(11):e80090 10.1371/journal.pone.0080090 24278244 PMC3836953

[7] Williams HF, Vaiyapuri R, Gajjeraman P, Hutchinson G, Gibbins JM, Bicknell AB, et al Challenges in diagnosing and treating snakebites in a rural population of Tamil Nadu, India: The views of clinicians. Toxicon. 2017;130:44–6. 10.1016/j.toxicon.2017.02.025 28238804

[8] Casewell NR, Wagstaff SC, Wüster W, Cook DA, Bolton FM, King SI, et al Medically important differences in snake venom composition are dictated by distinct postgenomic mechanisms. Proceedings of the National Academy of Sciences. 2014;111(25):9205–10. 10.1073/pnas.1405484111 PMC4078820 24927555

[9] Fry BG, Winkel KD, Wickramaratna JC, Hodgson WC, Wüster W. Effectiveness of snake antivenom: species and regional venom variation and its clinical impact. Journal of Toxicology: Toxin Reviews. 2003;22(1):23–34.

[10] Modahl CM, Mukherjee AK, Mackessy SP. An analysis of venom ontogeny and prey-specific toxicity in the Monocled Cobra (Naja kaouthia). Toxicon. 2016;119:8–20. 10.1016/j.toxicon.2016.04.049 27163885

[11] Wray KP, Margres MJ, Seavy M, Rokyta DR. Early significant ontogenetic changes in snake venoms. Toxicon. 2015;96:74–81. 10.1016/j.toxicon.2015.01.010 25600640

[12] Gutiérrez JM, Calvete JJ, Habib AG, Harrison RA, Williams DJ, Warrell DA. Snakebite envenoming. Nature Reviews Disease Primers. 2017;3:nrdp201763. 10.1038/nrdp.2017.79 28980622

[13] Vaiyapuri S, Wagstaff SC, Harrison RA, Gibbins JM, Hutchinson EG. Evolutionary analysis of novel serine proteases in the venom gland transcriptome of Bitis gabonica rhinoceros. PLoS One. 2011;6(6):e21532 10.1371/journal.pone.0021532 21731776 PMC3123349

[14] Vaiyapuri S, Harrison RA, Bicknell AB, Gibbins JM, Hutchinson G. Purification and functional characterisation of rhinocerase, a novel serine protease from the venom of Bitis gabonica rhinoceros. PLoS One. 2010;5(3):e9687 10.1371/journal.pone.0009687 20300193 PMC2837349

[15] Silva A, Johnston C, Kuruppu S, Kneisz D, Maduwage K, Kleifeld O, et al Clinical and Pharmacological Investigation of Myotoxicity in Sri Lankan Russell’s Viper (Daboia russelii) Envenoming. PLoS neglected tropical diseases. 2016;10(12):e0005172 10.1371/journal.pntd.0005172 27911900 PMC5135039

[16] Gutiérrez JM, Rucavado A, Chaves F, Díaz C, Escalante T. Experimental pathology of local tissue damage induced by Bothrops asper snake venom. Toxicon. 2009;54(7):958–75. 10.1016/j.toxicon.2009.01.038 19303033

[17] Jayawardana S, Gnanathasan A, Arambepola C, Chang T. Chronic musculoskeletal disabilities following snake envenoming in Sri Lanka: a population-based study. PLoS neglected tropical diseases. 2016;10(11):e0005103 10.1371/journal.pntd.0005103 27814368 PMC5096692

[18] Collins CA, Olsen I, Zammit PS, Heslop L, Petrie A, Partridge TA, et al Stem cell function, self-renewal, and behavioral heterogeneity of cells from the adult muscle satellite cell niche. Cell. 2005;122(2):289–301. 10.1016/j.cell.2005.05.010 16051152

[19] Caldwell C, Mattey D, Weller R. Role of the basement membrane in the regeneration of skeletal muscle. Neuropathology and applied neurobiology. 1990;16(3):225–38. 2402330 10.1111/j.1365-2990.1990.tb01159.x

[20] Gutiérrez JMa, Ownby CL. Skeletal muscle degeneration induced by venom phospholipases A 2: insights into the mechanisms of local and systemic myotoxicity. Toxicon. 2003;42(8):915–31. 10.1016/j.toxicon.2003.11.005 15019491

[21] Ownby CL, Cameron D, Tu A. Isolation of myotoxic component from rattlesnake (Crotalus viridis viridis) venom. Electron microscopic analysis of muscle damage. The American journal of pathology. 1976;85(1):149 970437 PMC2032543

[22] Duchen L, Excell BJ, Patel R, Smith B. Changes in motor end-plates resulting from muscle fibre necrosis and regeneration: a light and electron microscopic study of the effects of the depolarizing fraction (cardiotoxin) of Dendroaspis jamesoni venom. Journal of the neurological sciences. 1974;21(4):391–417. 4822123 10.1016/0022-510x(74)90041-0

[23] Bjarnason JB, Fox JW. Hemorrhagic metalloproteinases from snake venoms. Pharmacology & therapeutics. 1994;62(3):325–72. 7972338 10.1016/0163-7258(94)90049-3

[24] Baldo C, Jamora C, Yamanouye N, Zorn TM, Moura-da-Silva AM. Mechanisms of vascular damage by hemorrhagic snake venom metalloproteinases: tissue distribution and in situ hydrolysis. PLoS neglected tropical diseases. 2010;4(6):e727 10.1371/journal.pntd.0000727 20614020 PMC2894137

[25] Pinto AF, Terra RM, Guimaraes JA, Fox JW. Mapping von Willebrand factor A domain binding sites on a snake venom metalloproteinase cysteine-rich domain. Archives of biochemistry and biophysics. 2007;457(1):41–6. 10.1016/j.abb.2006.10.010 17118332

[26] Fox JW, Serrano SMT. Structural considerations of the snake venom metalloproteinases, key members of the M12 reprolysin family of metalloproteinases. Toxicon. 2005;45(8):969–85. 10.1016/j.toxicon.2005.02.012 15922769

[27] Clissa PB, Lopes-Ferreira M, Della-Casa MS, Farsky SHP, Moura-da-Silva AM. Importance of jararhagin disintegrin-like and cysteine-rich domains in the early events of local inflammatory response. Toxicon. 2006;47(5):591–6. 10.1016/j.toxicon.2006.02.001 16564063

[28] Ferreira BA, Deconte SR, de Moura FBR, Tomiosso TC, Clissa PB, Andrade SP, et al Inflammation, angiogenesis and fibrogenesis are differentially modulated by distinct domains of the snake venom metalloproteinase jararhagin. International Journal of Biological Macromolecules. 2018;119:1179–87. 10.1016/j.ijbiomac.2018.08.051 30102981

[29] Siigur E, Tonismagi K, Trummal K, Samel M, Vija H, Subbi J, et al Factor X activator from Vipera lebetina snake venom, molecular characterization and substrate specificity. Biochimica et biophysica acta. 2001;1568(1):90–8. 11731090 10.1016/s0304-4165(01)00206-9

[30] Yamada D, Sekiya F, Morita T. Prothrombin and factor X activator activities in the venoms of viperidae snakes. Toxicon. 1997;35(11):1581–9. 9428105 10.1016/s0041-0101(97)00043-3

[31] Gleason ML, Odell GV, Ownby CL. Isolation and biological activity of viriditoxin and a viriditoxin variant from Crotalus viridis viridis venoms. Journal of Toxicology: Toxin Reviews. 1983;2(2):235–65.

[32] Hernández R, Cabalceta C, Saravia-Otten P, Chaves A, Gutiérrez JM, Rucavado A. Poor regenerative outcome after skeletal muscle necrosis induced by Bothrops asper venom: alterations in microvasculature and nerves. PLoS One. 2011;6(5):e19834 10.1371/journal.pone.0019834 21629691 PMC3101212

[33] Vaiyapuri S, Hutchinson EG, Ali MS, Dannoura A, Stanley RG, Harrison RA, et al Rhinocetin, a venom-derived integrin-specific antagonist inhibits collagen-induced platelet and endothelial cell functions. Journal of Biological Chemistry. 2012;287(31):26235–44. 10.1074/jbc.M112.381483 22689571 PMC3406708

[34] Ravishankar D, Salamah M, Attina A, Pothi R, Vallance TM, Javed M, et al Ruthenium-conjugated chrysin analogues modulate platelet activity, thrombus formation and haemostasis with enhanced efficacy. Scientific reports. 2017;7(1):5738 10.1038/s41598-017-05936-3 28720875 PMC5515887

[35] Vaiyapuri S, Roweth H, Ali MS, Unsworth AJ, Stainer AR, Flora GD, et al Pharmacological actions of nobiletin in the modulation of platelet function. British journal of pharmacology. 2015;172(16):4133–45. 10.1111/bph.13191 25988959 PMC4543618

[36] Vaiyapuri S, Sage T, Rana RH, Schenk MP, Ali MS, Unsworth AJ, et al EphB2 regulates contact-dependent and independent signalling to control platelet function. Blood. 2014:blood-2014–06-585083. 10.1182/blood-2014-06-585083 PMC4304116 25370417

[37] Otto A, Schmidt C, Luke G, Allen S, Valasek P, Muntoni F, et al Canonical Wnt signalling induces satellite-cell proliferation during adult skeletal muscle regeneration. Journal of cell science. 2008;121(17):2939–50. 18697834 10.1242/jcs.026534

[38] Masuda S, Hayashi H, Araki S. Two vascular apoptosis‐inducing proteins from snake venom are members of the metalloprotease/disintegrin family. European journal of biochemistry. 1998;253(1):36–41. 9578458 10.1046/j.1432-1327.1998.2530036.x

[39] Allamand V, Briñas L, Richard P, Stojkovic T, Quijano-Roy S, Bonne G. ColVI myopathies: where do we stand, where do we go? Skeletal muscle. 2011;1(1):30. 21943391 10.1186/2044-5040-1-30 PMC3189202

[40] Igarashi T, Araki S, Mori H, Takeda S. Crystal structures of catrocollastatin/VAP2B reveal a dynamic, modular architecture of ADAM/adamalysin/reprolysin family proteins. FEBS letters. 2007;581(13):2416–22. 10.1016/j.febslet.2007.04.057 17485084

[41] Kikushima E, Nakamura S, Oshima Y, Shibuya T, Miao JY, Hayashi H, et al Hemorrhagic activity of the vascular apoptosis-inducing proteins VAP1 and VAP2 from Crotalus atrox. Toxicon. 2008;52(4):589–93. 10.1016/j.toxicon.2008.06.027 18657564

[42] Zhou Q, Smith J, Grossman M. Molecular cloning and expression of catrocollastatin, a snake-venom protein from Crotalus atrox (western diamondback rattlesnake) which inhibits platelet adhesion to collagen. Biochemical Journal. 1995;307(2):411–7. 7733877 10.1042/bj3070411 PMC1136664

[43] De Luca M, Ward CM, Ohmori K, Andrews RK, Berndt MC. Jararhagin and jaracetin: novel snake venom inhibitors of the integrin collagen receptor, alpha 2 beta 1. Biochemical and biophysical research communications. 1995;206(2):570–6. 7530003 10.1006/bbrc.1995.1081

[44] Zhou Q, Smith JB, Grossman MH. Molecular cloning and expression of catrocollastatin, a snake-venom protein from Crotalus atrox (western diamondback rattlesnake) which inhibits platelet adhesion to collagen. The Biochemical journal. 1995;307 (Pt 2):411–7. 7733877 10.1042/bj3070411 PMC1136664

[45] Herrera C, Escalante T, Voisin M-B, Rucavado A, Morazán D, Macêdo JKA, et al Tissue localization and extracellular matrix degradation by PI, PII and PIII snake venom metalloproteinases: clues on the mechanisms of venom-induced hemorrhage. PLoS neglected tropical diseases. 2015;9(4):e0003731 10.1371/journal.pntd.0003731 25909592 PMC4409213

[46] Moura-da-Silva AM, Ramos OH, Baldo C, Niland S, Hansen U, Ventura JS, et al Collagen binding is a key factor for the hemorrhagic activity of snake venom metalloproteinases. Biochimie. 2008;90(3):484–92. 10.1016/j.biochi.2007.11.009 18096518

[47] Lomonte B, Gutiérrez J. Phospholipases A2 From Viperidae Snake Venoms: How do They Induce Skeletal Muscle Damage? 2011 647–59 p. 24061112

[48] Dubovskii PV, Utkin YN. Cobra cytotoxins: structural organization and antibacterial activity. Acta naturae. 2014;6(3):11–8. 25349711 PMC4207557

[49] O'Brien J, Lee S-H, Gutiérrez JM, Shea KJ. Engineered nanoparticles bind elapid snake venom toxins and inhibit venom-induced dermonecrosis. PLoS neglected tropical diseases. 2018;12(10):e0006736–e. 10.1371/journal.pntd.0006736 30286075 PMC6171825

[50] Gutiérrez JM, León G, Rojas G, Lomonte B, Rucavado A, Chaves F. Neutralization of local tissue damage induced by Bothrops asper (terciopelo) snake venom. Toxicon. 1998;36(11):1529–38. 9792169 10.1016/s0041-0101(98)00145-7

[51] Chen J, Liaw S, Bullard M, Chiu T. Treatment of poisonous snakebites in northern Taiwan. Journal of the Formosan Medical Association = Taiwan yi zhi. 2000;99(2):135–9. 10770028

[52] Howes J-M, Theakston RDG, Laing G. Neutralization of the haemorrhagic activities of viperine snake venoms and venom metalloproteinases using synthetic peptide inhibitors and chelators. Toxicon. 2007;49(5):734–9. 10.1016/j.toxicon.2006.11.020 17196631

[53] Rucavado A, Escalante T, Gutiérrez JMa. Effect of the metalloproteinase inhibitor batimastat in the systemic toxicity induced by Bothrops asper snake venom: understanding the role of metalloproteinases in envenomation. Toxicon. 2004;43(4):417–24. 10.1016/j.toxicon.2004.01.016 15051405

[54] Ainsworth S, Slagboom J, Alomran N, Pla D, Alhamdi Y, King SI, et al The paraspecific neutralisation of snake venom induced coagulopathy by antivenoms. Communications Biology. 2018;1(1):34. 30271920 10.1038/s42003-018-0039-1 PMC6123674

