# Supplementary material for: Mechanisms underpinning the permanent muscle damage induced by snake venom metalloprotease
Source: PLoS Negl Trop Dis. 2019 Jan 29;13(1):e0007041. doi: 10.1371/journal.pntd.0007041 (PMC6368331; doi:10.1371/journal.pntd.0007041)
Supplement: S1 Table — (DOCX) [file pntd.0007041.s001.docx]

**Supplementary Table 1**

| Antigen | Species | Dilution | Supplier |
| --- | --- | --- | --- |
| Pax7 | Mouse | 1:1 | DSHB-Pax7 |
| MyoD | Rabbit | 1:200 | Santa Cruz sc-760 |
| Laminin | Rabbit | 1:200 | Sigma Aldrich L9393 |
| MYH3 | Mouse | 1:200 | Santa Cruz sc-53091 |
| Collagen IV | Rabbit | 1:200 | Abcam ab6586 |
| CD31 | Rat | 1:40 | AbD serotec MCA2388 |
| F4/80 | Rat | 1:100 | Bio-RAD MCA4978 |
| Dystrophin | Rabbit | 1:200 | Abcam 15277 |
| nNOS | Rabbit | 1:200 | Santa Cruz, 648 |
| CAMP | Sheep | 1:200 | In house preparation |
| Alexa fluor 488 anti-mouse | Goat | 1:200 | Life Technologies A11029 |
| Alexa fluor 488 anti-rabbit | Goat | 1:200 | Life Technologies A11034 |
| Alexa fluor 594 anti-rabbit | Goat | 1:200 | Life Technologies A11037 |
| Alexa fluor 488 anti-rat | Goat | 1:200 | Life Technologies A11006 |
| Alexa Fluor 546 anti-sheep | Donkey | 1:200 | Invitrogen A-21098 |
